# How to manage synchronous endometrial and ovarian cancer patients?

**DOI:** 10.1186/s12885-021-08220-w

**Published:** 2021-05-01

**Authors:** Wonkyo Shin, Sang-Yoon Park, Sokbom Kang, Myong Cheol Lim, Sang-Soo Seo

**Affiliations:** 1 Center for Gynecologic Cancer, Graduate School of Cancer Science and Policy, National Cancer Center, 323 Ilsan-ro, Ilsandong-gu, Goyang-si, Gyeonggi-do 10408 Republic of Korea; 2 Department of Obstetrics & Gynecology, Chungnam national university sejong hospital, Sejong, Republic of Korea; 3 Common Cancer Branch, Research Institute Graduate School of Cancer Science and Policy, National Cancer Center, Goyang, Republic of Korea; 4 Precision Medicine Branch, Graduate School of Cancer Science and Policy, National Cancer Center, Goyang, Republic of Korea; 5 Department of Cancer Control & Population Health, Graduate School of Cancer Science and Policy, National Cancer Center, Goyang, Republic of Korea; 6 Center for Clinical Trials, Graduate School of Cancer Science and Policy, National Cancer Center, Goyang, Republic of Korea; 7 Cancer Healthcare Research Branch, Graduate School of Cancer Science and Policy, National Cancer Center, Goyang, Republic of Korea

**Keywords:** Endometrial neoplasms, Synchronous cancer, Ovarian cancer, Metastasis, Prognosis

## Abstract

**Backgrounds:**

We aimed to evaluate the prognosis in patients with synchronous endometrial and ovarian cancer (SEOC) by comparing the differences between double primary cancer (DPC) and metastatic cancer (MC).

**Methods:**

The medical records of 47 patients diagnosed synchronously with endometrial and ovarian cancer between January 2006 and December 2018 were retrospectively reviewed. Twenty-eight and 19 patients were diagnosed with DPC and MC, respectively. Demographics, recurrence-free survival (RFS), and 5-year overall survival (OS) were compared. The clinical factors affecting survival were evaluated using univariate and multivariate analyses.

**Results:**

The demographics were not different between both groups. Endometrioid histology and the International Federation of Gynecology and Obstetrics grade were higher in the MC group than in the DPC group (42.1% vs. 10.7%; *P* = 0.018, *P* = 0.002, respectively). The ratio of post-operative adjuvant therapy was not different in both groups. Recurrence occurred in five patients with DPC and seven with MC. The difference in RFS was not significantly different (*P* = 0.131) but the OS was different between both groups (*P* = 0.020). Histology and para-aortic lymph node metastasis were associated wtih RFS in univariate analysis, but no difference was found in multivariate analysis.

**Conclusions:**

Although DPC patients had longer OS, multivariate analysis did not identify any influential factors. Focus should be placed on defining the appropriate adjuvant treatment for high-risk patients, which will improve prognosis, rather than on discriminating between DPC and MC.

**Supplementary Information:**

The online version contains supplementary material available at 10.1186/s12885-021-08220-w.

## Backgrounds

Endometrial cancer has the highest incidence among gynecological cancers in Western countries [1]. In Korea, the diagnosis of endometrial cancer has been steadily increasing over the last 10 years [2]. Endometrial cancer is usually diagnosed at an early stage because patients present for consultations at the hospital with abnormal vaginal bleeding or discharge. The diagnosis is usually confirmed either by biopsy with endometrial curettage or hysteroscopy. The disease status is confirmed through imaging (computed tomography [CT] scan or magnetic resonance imaging [MRI]), and then the treatment method is determined. If the tumor is resectable, a surgery involving total hysterectomy, bilateral salpingo-oophorectomy, pelvic and para-aortic lymph node (LN) dissection is performed. The adjuvant treatment option is chosen based on the pathologic report. Cancer may also be found incidentally in the ovaries in about 7% of the endometrial cancer patients [3]. The tumor stages in such cases are dependent on whether it is a metastasis of an endometrial cancer, metastasis of an ovarian cancer, or a co-occurrence of both cancers in the ovaries and endometrium.

In 1985, Ulbright and Roth proposed criteria for distinguishing metastatic cancer (MC) from double primary cancer (DPC) in such cases [4]. In 1998, Scully and Young proposed more detailed diagnostic criteria [5]. Since the above criteria are widely used, there are many studies comparing the characteristics and prognoses in these two groups (MC and DPC) [6–17]. A prior study suggested that the prognosis was poor when metastasis involved other sites in addition to the uterus and ovaries and when there was no distinction between the DPC and MC [7]. Endometrioid histology has been shown to have a better prognosis than non-endometrioid histology [8, 9]. Compared to DPC, MC has a poorer prognosis with cervical invasion, a large tumor size, and high-grade histology [10]. Early-stage DPC showed a good prognosis in a study conducted only on DPC patients [12]. Song et al. showed the association of the initial CA-125 level and ovarian stage with DPC survival [13], while Jain et al. showed the association between lymphovascular invasion and DPC survival [14]. When comparing DPC with endometrial cancer, prognosis is not inferior than that in endometrial cancer [15]. However, the prognosis in patients with cervical invasion, LN metastasis, and peritoneal dissemination, regardless of DPC or MC, is poor [17]. Overall, the prognosis is good in early-stage DPC. It is difficult to accurately differentiate between DPC and MC based on these classical criteria or morphological differences including histopathology, size, and spread to adjacent organs.

On the one hand, in 2016, two independent studies reported that most synchronous endometrial and ovarian cancers (SEOCs) were single primary tumors with metastases; this was evaluated using massively parallel sequencing [18, 19]. Accordingly, Chao et al. analyzed 16 SEOC patients with massively parallel sequencing and copy number analysis [20]. These studies support the fact that SEOC is a metastatic disease and not a DPC. However, it is difficult to apply these results in real-world clinical settings, because of high cost and long time needed for analysis.

On the other hand, categorization of the tumor as either DPC or MC is important for accurate diagnosis. However, determining the appropriate treatment modality for patients diagnosed with either DPC or MC is more important. If the diagnosis is DPC, each organ’s tumor is staged as IA; if the diagnosis is MC, the tumor is staged as IIIA based on the endometrial cancer staging or IIA based on the ovaries. It is thus important to know if the cancer occurs synchronously in the ovaries and endometrium or metastasizes from one organ to another. This helps to select patients who need adjuvant treatment, be it in the form of chemotherapy or radiotherapy. Therefore, we analyzed and compared the baseline characteristics of DPC and MC patients and analyzed the risk factors for recurrence.

## Methods

### Study population

The electronic medical records of patients who were newly diagnosed with endometrial cancer and ovarian cancer at the National Cancer Center in South Korea between January 2006 and December 2018 were reviewed. Forty-seven patients who had been diagnosed and treated at our center were included in the analysis. Patient clinical characteristics, including age at diagnosis, tumor size, radicality of hysterectomy, LN dissection, lymphovascular invasion, endocervical invasion, International Federation of Gynecology and Obstetrics (FIGO) stage and grade, histology, surgical procedure, and the records of post-operative adjuvant chemotherapy and radiotherapy, were collected through an electronic search of the center’s medical records.

### Classification of DPC and MC

Ulbright and Roth classified the MC as followed criteria. Metastatic carcinoma was diagnosed based on a multinodular ovarian pattern as a major criterion with two or more of the following as minor criteria: small (< 5 cm) ovary(ies), bilateral ovarian involvement, deep myometrial invasion, vascular invasion, and tubal lumen involvement. The more extensive and detailed Scully and Young criteria was reviewed in additional file 1. The patients’ pathology in this study was differentiated as DPC versus MC using Scully and Young criteria.

### Statistical analysis

Correlations of variables were assessed using Fisher’s exact test or Student’s *t*-test. The five-year overall survival (OS) and recurrence-free survival (RFS) were estimated using the Kaplan–Meier method, and the significance of differences was determined using log-rank tests. Univariate and multivariate Cox regression analyses were performed to identify the patient characteristics associated with prognosis. Hazard ratios (HR) were calculated. *P*-values < 0.05 were considered significant.

## Results

**Fig. 1:**
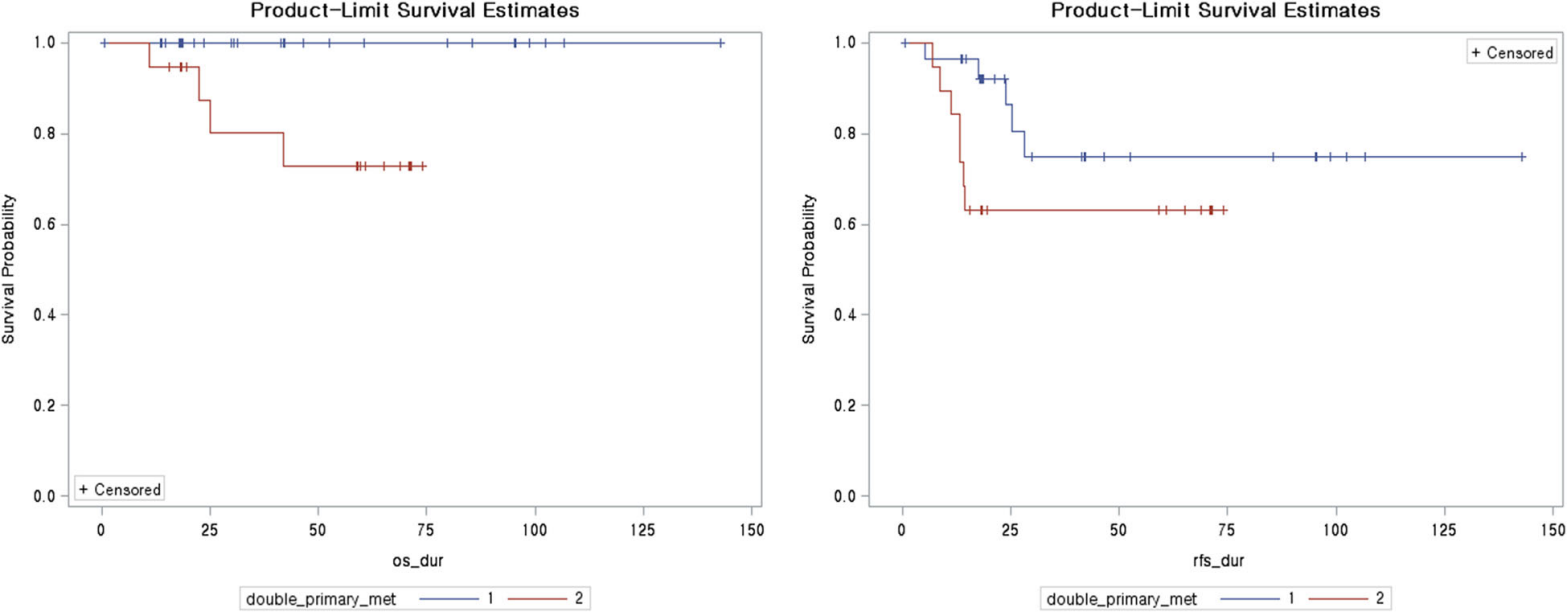
Kaplan-Meier overall survival and recurrence curves in DPC and MC. DPC, double primary cancer; MC, metastatic cancer

Among the total of 47 patients, 28 were diagnosed with DPC, and 19 with MC. The demographics of the two groups are compared in Table 1. Surgical approach, LN dissection, lymph node pathology, lymphovascular invasion, and endocervical invasion were not different between the two groups. The endometrial cancer and ovarian cancer FIGO grades were significantly higher in the MC group than in the DPC group (*P* = 0.001, *P* = 0.026, respectively). Endometrioid histology of the endometrium was higher in the DPC group (*P* = 0.018). Post-operative adjuvant therapy in the two groups was not different. The Kaplan–Meier survival analyses of RFS and 5-year OS are shown in Fig. 1. Differences in RFS were not statistically different (*P* = 0.131), but the difference in OS was significant (*P* = 0.020). In univariate analysis, endometrioid histology of the endometrium (*P* = 0.002) and ovary (*P* = 0.016) showed lower recurrence than other histologies, and para-aortic lymph node metastasis was related to recurrence (*P* = 0.026). Lymphovascular invasion, endocervical invasion, and FIGO grade were not related to recurrence. No clinical factors were found in multivariate analysis. Only endometrioid histology compared with non-endometrioid histology showed a trend to better OS (HR = 0.09, *P* = 0.035) (Table 2). Twelve patients (5 DPC and 7 MC) showed disease recurrence. The detailed clinical characteristics of recurrent patients are descripted in Table 3. There were no specific different clinical factors, four patients died in the MC group and no patients died in the DPC group. The comparison of DPC histology findings is presented in Additional file 2.

**Table 1:** Baseline characteristics of patients

Variables	Total (*N* = 47)	DP (*N* = 28)	Meta (*N* = 19)	*P*-value
Age (med, min-max)	52 (24–70)	50 (24–69)	54 (39–70)	0.263
Approach				1.000
Laparoscopy	9 (19.1)	5 (17.9)	4 (21.1)	
Laparotomy	38 (80.9)	23 (82.1)	15 (78.9)	
PLND				0.685
No	7 (14.9)	5 (17.9)	2 (10.5)	
Yes	40 (85.1)	23 (82.1)	17 (89.5)	
PALND				1.000
No	12 (25.5)	7 (25.0)	5 (26.3)	
Yes	35 (74.5)	21 (75.0)	14 (73.7)	
LVS I				0.417
No	40 (85.1)	25 (89.3)	15 (78.9)	
Yes	7 (14.9)	3 (10.7)	4 (21.1)	
Endocervix-invasion				0.381
No	42 (89.4)	26 (92.9)	16 (84.2)	
Yes	5 (10.6)	2 (7.1)	3 (15.8)	
Pelvic_peritoneum_invasion				0.485
No	36 (76.6)	20 (71.4)	16 (84.2)	
Yes	11 (23.4)	8 (28.6)	3 (15.8)	
Endometrial FIGO stage				**<.001**
1	30 (63.8)	24 (85.8)	6 (31.6)	
2	2 (4.3)	2 (7.1)	0 (0.0)	
3	12 (25.5)	2 (7.1)	10 (52.6)	
4	3 (6.4)	0 (0.0)	3 (15.8)	
Ovarian FIGO stage	miss = 12			**0.038**
1	19 (54.2)	16 (57.1)	3 (42.9)	
2	8 (22.9)	8 (28.6)	0 (0.0)	
3	7 (20.0)	4 (14.3)	3 (42.9)	
4	1 (2.9)	0 (0.0)	1 (14.2)	
Endometrial histology				**0.018**
Non-endometrioid	11 (23.4)	3 (10.7) 2 serous 1 clear cell	8 (42.1) 5 serous 1 clear 1 mixed 1 carcinosarcoma	
Endometrioid	36 (76.6)	25 (89.3)	11 (57.9)	
Ovarian histology				0.210
Non-endometrioid	22 (46.8)	11 (39.3) 5 serous 2 clear 2 seromucinous 1 mucinous 1 carcinosarcoma	11 (57.9) 8 serous 2 mixed 1 carcinosarcoma	
Endometrioid	25 (53.2)	17 (60.7)	8 (42.1)	
Endometrial FIGO grade	miss = 8			**0.002**
1	17 (43.6)	16 (61.5)	1 (7.7)	
2	11 (28.2)	6 (23.1)	5 (38.5)	
3	11 (28.2)	4 (15.4)	7 (53.8)	
Ovarian grade	miss = 7			0.056
1	15 (37.5)	13 (50.0)	2 (14.3)	
2	13 (32.5)	8 (30.8)	5 (35.7)	
3	12 (30.0)	5 (19.2)	7 (50.0)	
Adjuvant chemotherapy				0.685
No	7 (14.9)	5 (17.9)	2 (10.5)	
Yes	40 (85.1)	23 (82.1)	17 (89.5)	
Adjuvant radiotherapy				1.000
No	42 (89.4)	25 (89.3)	17 (89.5)	
Yes	5 (10.6)	3 (10.7)	2 (10.5)	

*DP* Double primary, *PALND* Para-aortic lymph node dissection, *PLND* Pelvic lymph node dissection

**Table 2:** Cox-regression analysis of risk factors

Variables	Overall survival			Recurrence-free survival		
	N (event)	HR (95% CI)	***P***-value	N (event)	HR (95% CI)	***P***-value
Age	47 (4)	1.05 (0.93–1.18)	0.428	47 (12)	1.05 (0.98–1.11)	0.173
Pelvic_LN_pathology						
No	41 (3)	1		41 (9)	1	
Yes	6 (1)	3.57 (0.36–35.05)	0.275	6 (3)	3.86 (0.96–15.46)	0.057
paraaortic_LN						
No	42 (3)	1		42 (9)	1	
Yes	5 (1)	3.80 (0.39–36.90)	0.250	5 (3)	4.84 (1.21–19.41)	**0.026**
LVS						
No	40 (3)	1		40 (11)	1	
Yes	7 (1)	2.29 (0.24–22.13)	0.474	7 (1)	0.54 (0.07–4.18)	0.554
Endocervix-invasion						
No	42 (4)	1		42 (11)	1	
Yes	5 (0)			5 (1)	0.60 (0.08–4.64)	0.623
Pelvic peritoneum invasion						
No	36 (2)	1		36 (8)	1	
Yes	11 (2)	3.17 (0.44–22.96)	0.253	11 (4)	1.63 (0.49–5.41)	0.427
Stage_EM						
1 + 2	32 (2)	1		32 (9)	1	
3 + 4	15 (2)	2.60 (0.36–18.54)	0.342	15 (3)	0.71 (0.19–2.63)	0.610
Stage_OV						
1 + 2	27 (0)	1		27 (3)	1	
3 + 4	8 (2)			8 (6)	12.16 (2.87–51.59)	**0.001**
Histology_EM						
Non-endometrioid	11 (3)	1		11 (7)	1	
Endometrioid	36 (1)	0.09 (0.01–0.84)	**0.035**	36 (5)	0.15 (0.05–0.49)	**0.002**
Histology_OV						
Non-endometrioid	22 (4)	1		22 (9)	1	
Endometrioid	25 (0)			25 (3)	0.20 (0.05–0.73)	**0.016**
Grade_EM						
1	17 (1)	1		17 (3)	1	
2 + 3	22 (1)	0.81 (0.05–13.01)	0.884	22 (2)	0.59 (0.1–3.55)	0.567
Grade_OV						
1	15 (1)	1		15 (4)	1	
2 + 3	25 (2)	1.22 (0.11–13.45)	0.873	25 (5)	0.84 (0.23–3.14)	0.798
Adjuvant CTx						
No	7 (1)	1		7 (1)	1	
Yes	40 (3)	0.47 (0.05–4.54)	0.516	40 (11)	1.95 (0.25–15.16)	0.521
Adjuvant RTx						
No	42 (4)	1		42 (10)	1	
Yes	5 (0)			5 (2)	1.35 (0.30–6.17)	0.700

*CI* Confidence interval, *CTx* Chemotherapy, *RTx* Radiotherapy, *EM* Endometrium, *OV *Ovary, *HR* Hazard ratio, *LN* Lymph nodes

**Table 3:** Clinical characteristics of recurrent patients

Age	Pelvic LN metastasis	Paraaortic LN metastasis	LVS I	Endocervix invasion	Metastasis to other sites	Stage EM	Stage OV	DPC or MC	Histology_EM	Histology_OV	Follow up period	Death	RFS	Adjuvant CTx	Adjuvant RTx	Recurrence site	Histology and origin of recurrence
57	yes	yes	no	no	no	IB	IIIC	DPC	clear cell	serous, Gr3	81	no	18	yes	no	Mediastinal LN	Serous, ovary
56	no	no	no	no	yes (CPLN)	IA	IV	DPC	endometrioid, Gr1	serous	60	no	9	yes	no	Peritoneal seeding	Serous, ovary
57	no	no	no	no	yes (broad lig)	IA	IIA	DPC	endometrioid, Gr1	endometrioid, Gr1	32	no	29	yes	yes	Paracolic gutter	Endometrioid, ovary
60	no	no	no	yes	no	II	IA	DPC	serous	endometrioid, Gr1	24	no	28	yes	yes	Vaginal stump	Serous, endometrium
50	no	no	no	no	no	IA	IA	DPC	endometrioid, Gr2	seromucinous	26	no	7	yes	no	Peritoneal seeding	Endometrioid, endometrium
60	no	no	no	no	no	IIIA	n/a	MC	carcinosarcoma	carcinosarcoma	17	yes	14	yes	no	Para-aortic LN	Carcinosarcoma
52	yes	yes	yes	no	yes (CPLN, broad lig)	IVB	n/a	MC	serous, Gr3	serous, Gr3	7	yes	5	yes	no	Peritoneal seeding	Serous
39	no	no	no	no	no	IIIA	n/a	MC	serous, Gr3	serous, Gr3	15	no	13	yes	no	Peritoneal seeding	Serous
55	no	no	no	no	no	IA	IIIC	MC	serous	serous, Gr3	40	yes	15	yes	no	Peritoneal seeding	Serous
60	no	no	no	no	yes (liver capsule)	IB	IIIB	MC	endometrioid, Gr1	endometrioid, Gr1	61	no	24	yes	no	Peritoneal seeding	Endometrioid
52	no	no	no	no	yes (broad lig, omentum)	IA	IIIC	MC	serous	serous, Gr3	22	yes	13	yes	no	Common iliac LN	Serous
56	yes	yes	no	no	yes (salpinx, broad lig, LN meta)	IA	IIIB	MC	serous	serous, Gr3	59	no	13	yes	no	Peritoneal seeding	Serous

*CPLN* Cardio phrenic lymph nodes, *DPC* Double primary cancer, *EM* Electron microscopy, *MC* Metastatic cancer, *LN* Lymph nodes, *LVS* Lymphatic vessels

## Discussion

The rate at which cancer is found synchronously in the ovaries and endometrium is approximately 3–10% [21]. The Ulbright and Roth criteria proposed in 1986 help in differentiating DPC from MC. We included both DPC and MC patients in our study. However, the clinical factors and survival rate in patients in the two groups were not significantly different. Endometrioid histology of the endometrium (*P* = 0.002) and ovaries (*P* = 0.016) and para-aortic lymph node metastasis (*P* = 0.026) were the risk factors for recurrence, regardless of either DPC or MC.

Almost all SEOCs were evaluated as single primary tumors with metastasis using next-generation sequencing (NGS) in two recently published studies [18–20]. NGS is an accepted accurate diagnostic tool in various carcinomas, and it is being used increasingly for the diagnosis and treatment of endometrial and ovarian cancers. Genetic analysis using NGS may be accurate in evaluating the characteristics of cancer. However, there was no significant difference in the clinical factors or prognosis between the two groups in those studies. This led to the question of the necessity of classifying two groups and the use of NGS.

The tumor is staged as IA if it is DPC. If it is MC, it is staged as IIIA based on the endometrium or II based on the ovaries. If diagnosed as IA, no additional treatment is required. If diagnosed as IIIA or II, additional treatment is required. Using pathology in distinguishing between DPC and MC may lead to mis-staging; therefore, there are potential risks of wrong management of the patients.

Whether DPC or MC is diagnosed using NGS or pathology, only using the time difference, makes it difficult to determine whether the disease occurred concurrently in both organs or it had metastasized from one organ to the other through an unknown mechanism. Making an accurate differentiation between DPC and MC remains a problem even if the pathology and NGS results are the same. A successful cancer metastasis requires a series of sequential steps such as cancer cell migration, settlement, proliferation, vascularization, etc. This is an inefficient process for cancer cells. Furthermore, even if the NGS results are different, it is impossible to rule out the possibility of either a metastases or DPC. Several reports have shown the cases wherein the genomes of the tumor origin and the metastatic site were different [22–24]. Whether or not a clear-cut difference can be established between DPC and MC using various methods, it does not affect the necessity for the adjuvant treatment.

In this study, the clinical features of 12 patients (5 DPC and 7 MC) with recurrence were assessed, and these data are summarized in Table 3. A non-endometrioid histology and a high FIGO grade were mostly observed in MC. When cancer is diagnosed in the ovary and endometrium synchronously, whether the diagnosis of DPC or MC is made using pathology or NGS, the adjuvant treatment option is determined clinically by the risk factors of each individual patient. Therefore, it would be more important to determine the risk factors and the need for adjuvant treatment rather than how the diagnosis is made.

To increase the reliability of our findings, there is a need for a large multicenter study focusing on the identification of risk factors. This can help improve the prognosis, disease-free survival, and cure rate through aggressive treatment and strong surveillance in patients with synchronous ovarian and endometrial cancer with risk factors for recurrence.

## Conclusions

It is necessary to focus on defining the appropriate adjuvant treatment for high-risk patients, rather than discriminating between DPC and MC. Although, DPC patients had longer OS, multivariate analysis did not identify any influential factors in our retrospective study,

## Supplementary Information

**Additional file 1.** Criteria for categorizing double primary endometrial and ovarian tumors. Summarize the criteria of DPC by Scully and Young.

**Additional file 2.** Comparison of double primary tumor. Comparison of DPC by histology.

## Data Availability

The datasets used and/or analyzed during the current study are available from the corresponding author on reasonable request.

## Abbreviations

CT: Computed tomography
DPC: Double primary cancer
FIGO: Federation of Gynecology and Obstetrics
HR: Hazard ratios
LN: Lymph node
MC: Metastatic cancer
NGS: Next-generation sequencing
OS: Overall survival
RFS: Recurrence-free survival
SEOC: Synchronous endometrial and ovarian cancer.
