# Supplementary material for: How to manage synchronous endometrial and ovarian cancer patients?
Source: BMC Cancer. 2021 May 1;21:489. doi: 10.1186/s12885-021-08220-w (PMC8088669; doi:10.1186/s12885-021-08220-w)
Supplement: Supplementary file 1 — Additional file 1. Criteria for categorizing double primary endometrial and ovarian tumors. Summarize the criteria of DPC by Scully and Young. [file 12885_2021_8220_MOESM1_ESM.docx]

| Additional file 1. Criteria for categorizing double primary endometrial and ovarian tumors |
| --- |
| 1) Histological dissimilarity of the tumors  2) No or only superficial myometrial invasion of the endometrial tumor  3) No vascular space invasion of the endometrial tumor  4) Atypical endometrial hyperplasia additionally present  5) Absence of other evidence of spread of the endometrial tumor  6) Ovarian unilateral tumor  7) Ovarian tumor located in the ovarian parenchyma  8) No vascular space invasion, surface implants or predominant hilar location in ovary  9) Absence of other evidence of spread of the ovarian tumor  10) Ovarian endometriosis present  11) Different ploidy of DNA indices, if aneuploid, of the tumors  12) Dissimilar molecular genetic or karyotypic abnormalities in the tumors |
