# Supplementary material for: How to manage synchronous endometrial and ovarian cancer patients?
Source: BMC Cancer. 2021 May 1;21:489. doi: 10.1186/s12885-021-08220-w (PMC8088669; doi:10.1186/s12885-021-08220-w)
Supplement: Supplementary file 2 — Additional file 2. Comparison of double primary tumor. Comparison of DPC by histology. [file 12885_2021_8220_MOESM2_ESM.docx]

| Endometrium  Ovary | Endometrioid | Non-endometrioid |
| --- | --- | --- |
| Endometrioid | 16 | EM  1 serous |
| Non-endometrioid | Ovary  3 serous  2 clear cell  1 carcinosarcoma  2 seromucinous  1 mucinous | 1 OV serous  EM clear cell  1 OV/EM serous |

Additional file 2. Comparison of double primary tumor

EM, endometrial; OV, ovarian
